# In vitro analysis of a glass solder matrix on ATZ-ceramic-samples using human dental pulp cells and L929 mouse fibroblasts

**DOI:** 10.1186/s40729-026-00697-z

**Published:** 2026-06-24

**Authors:** Sandra Fuest, Moritz L. Dyck, Mustafa Becerikli, Andreas Schmoock, Anders Henningsen, Christian Moss, Robert Mau, Hermann Seitz, Reinhard E. Friedrich, Martin Gosau, Ralf Smeets, Levi Matthies

**Affiliations:** 1 Department of Oral and Maxillofacial Surgery, Division of Regenerative Orofacial Medicine, University Medical Center Hamburg-Eppendorf, 20246 Hamburg, Germany; 2 Department of Oral and Maxillofacial Surgery, University Medical Center Hamburg-Eppendorf, 20246 Hamburg, Germany; 3 Department of Oral and Maxillofacial Surgery, Karlsruhe Hospital, 76133 Karlsruhe, Germany; 4 Department of Plastic Surgery, BG University Hospital Bergmannsheil, Ruhr-University Bochum, 44789 Bochum, Germany; 5 Dental Laboratory Moss, 20097 Hamburg, Germany; 6 Chair of Microfluidics, University of Rostock, 18059 Rostock, Germany

**Keywords:** Dental implant, Ceramic implant, Zirconium, Glass solder, Glass matrix, Surface modification

## Abstract

**Purpose:**

Optimized surface properties are a pivotal factor for dental ceramic implants and overall implant success. After grit-blasting and spray-coating Aluminum Toughened Zirconia (ATZ) ceramic samples with a glass solder, the biological cell response was examined and compared to uncoated and solely grit-blasted samples of the same ceramic.

**Methods:**

In this study, a silica-based glass solder was evaluated for cytocompatibility, osteogenic differentiation, and hemocompatibility using L929 mouse fibroblasts and human dental pulp cells (HDPCs).

**Results:**

No toxic effects were observed for either coated or uncoated ATZ samples in direct or indirect tests, as assessed by live/dead staining. Differentiation and viability assays with HDPCs and L929 mouse fibroblasts showed no toxicity, and osteogenic differentiation was not impaired by the coating. Hemocompatibility testing with human whole blood revealed similar results for coated and uncoated ceramic specimens, with no hemolysis or adverse effects on standard hematological parameters.

**Conclusions:**

The glass solder coating was cytocompatible and hemocompatible under the tested in vitro conditions and did not impede osteogenic differentiation. These findings indicate that it can serve as a suitable substrate for the proliferation and spreading of L929 fibroblasts and HDPCs, with potential benefits for improving dental ceramic implant surfaces.

## Background

Titanium (Ti) implants, with their advantageous mechanical properties, high corrosion stability, established biocompatibility, and reliable osseointegration, have long been the gold standard in dental implantology [1]. They are widely recognized as a safe and effective therapeutic option [2]. However, the search for optimized biomaterials has spurred interest in ceramic implants as alternatives, particularly Alumina-Toughened Zirconia (ATZ) ceramics. Historically, Ti implants were considered mechanically superior to ceramics, but recent improvements in ceramic strength and toughness have reduced fracture rates of ATZ implants from 3.4% in 2004 to 0.2% in 2017 [3]. In 2022, the German national treatment guidelines for dental implants (including guidelines on titanium hypersensitivity) acknowledged one-piece ceramic implants as a viable alternative to established titanium systems [4, 5]. At that time, two-piece ceramic implants were not yet recommended due to limited long-term data, although recent studies on two-piece ceramic implant survival are promising [6].

Ceramic implants offer several advantages over titanium. With a similar micro roughness surface profile, ceramics are more esthetically appealing, more resistant to corrosion, and potentially immunologically favorable compared to titanium [7–11]. The grayish color of titanium can be problematic in visible regions, and even in posterior regions a metallic hue can shine through if gingival recession or thin tissue is present. Moreover, galvanic interactions between Ti and other metallic restorations have been reported, as have corrosion-related particle release despite the protective oxide layer on Ti surfaces [12–15]. From an immunological perspective, Ti implants have been associated with cases of intolerance, hypersensitivity, and other adverse reactions [1, 5, 16, 17]. Ti wear particles can elicit inflammatory responses by stimulating cytokines such as tumor necrosis factor alpha (TNF-α) and interleukin-1 (IL-1), leading to increased osteoclast activity and collagenase release. These changes have been linked to peri-implant inflammation and bone loss. Notably, Ti particles have also been detected in distant tissues and organs [18]. Due to these drawbacks, ceramic implants have been gaining attention in dental implantology. Studies have highlighted several benefits of ceramic implants: they have a bright tooth-like color, excellent biocompatibility, lower plaque accumulation, favorable soft-tissue interaction, good osseointegration, and superior tribological (wear) properties [19–22]. Ceramic implants are more resistant to corrosion than Ti and do not induce galvanic currents when paired with metallic superstructures or fillings [11, 12]. Immunologically, zirconia (zirconium dioxide) implants may be favorable: they release fewer particles into surrounding tissues than Ti, resulting in significantly lower TNF-α and RANKL (Receptor Activator of NF-κB Ligand) levels in vitro [23, 24]. Unlike Ti, ceramics have not had any confirmed hypersensitivity reactions reported to date [1, 16, 17, 25]. Ceramic implants also exhibit better soft-tissue responses, lower bacterial survival and adhesion on their surfaces, and favorable peri-implant health indices (bleeding on probing and probing pocket depth) compared to even natural teeth [4, 9, 19–21, 26, 27].

Despite these advantages, ceramic implants still show bone-to-implant contact (BIC) rates around 65–73%, approximately comparable to Ti [28]. The optimal surface topography for maximal protein adsorption and osteoblast attachment remains unknown [29]. It is well recognized that surface properties—both micro topography and chemistry—are critical for cell attachment and osseointegration [30–32]. Osteoblast and fibroblast progenitor adhesion, proliferation, and differentiation at the interface are pivotal for long-term implant stability. Consequently, extensive research has focused on modifying implant surfaces to improve BIC rates. Traditional surface modification methods for dental implants include ablative techniques (e.g. acid etching, sand/grit-blasting) and deposition coating techniques (e.g. dip-coating, plasma-spraying, or spray-coating with biomaterials). These methods do produce micro-rough surfaces, but they have limitations. In vitro studies have shown that human gingival fibroblasts align preferentially on substrates with regularly spaced micro-depressions (~ 1 μm in width and depth) [31]. It is noteworthy that, at present, ceramic implant surfaces (after modification) achieve osseointegration outcomes comparable to Ti [8–10]. Although numerous surface modification techniques for dental ceramics have been explored—such as sandblasting, acid etching, plasma spraying, and sol‑gel coatings—these conventional methods often produce irregular micro‑topographies and non‑uniform coating thicknesses. Such variability can lead to inconsistent cellular responses and uncertain long‑term adhesion between the coating and the ceramic substrate. Until today, there is limited evidence on glass‑based coatings for ATZ ceramics that are both biocompatible and technologically adaptable for precise application methods [33].

In this context, the present study aimed to evaluate a silica‑based glass solder layer applied via spray‑coating as an innovative surface modification for ATZ ceramics. Spray‑coating allows controlled, homogeneous deposition of the glass matrix on the ceramic surface while avoiding chemical residues associated with acid‑based treatments or the high thermal stress of plasma spraying. Furthermore, this glass solder formulation is compatible with additive manufacturing methods such as inkjet printing, opening future possibilities for creating defined micro‑patterns once its biocompatibility is established.

The objective of this study, therefore, was to investigate whether the glass solder coating is cytocompatible, hemocompatible, and permissive of osteogenic differentiation under standardized in vitro conditions, thereby providing a foundation for future functional surface design on ceramic dental implants.

## Methods

### Zirconium specimens

ATZ ceramic samples (Metoxit AG, Thayngen, Switzerland) were used in this study. According to DIN EN 60267, the material consists of 76% ZrO₂, 20% Al₂O₃, and 4% Y₂O₃. ATZ disks were manufactured with a diameter of 13 ± 0.3 mm and 1.5 mm thickness.

### Surface modification

Prior to coating, the ATZ samples were pre-conditioned by sandblasting with 110 µm alumina (Al₂O₃) grit at 2 bar pressure. The samples were then cleaned in 80% ethanol for 30 min and allowed to dry. A silica-based glass solder (Estetic Ceram AG, Triesen, Liechtenstein) was applied to the sample surfaces by spray-coating, followed by sintering at 1035 °C. This solder is an intermediate product used in the production of type 1, class 1 dental ceramics (compliant with DIN EN ISO 6872), composed of approximately SiO₂ 59%, Al₂O₃ 16%, K₂O 5.5%, and Na₂O 7%. Two cycles of coating and sintering were performed for each sample. After the second sintering, the glass-solder-coated surface was roughened with a light sandblasting (110 µm Al₂O₃ at 1 bar) and then cleaned with a steam jet. Control ATZ samples underwent the same grit-blasting procedures but remained uncoated. The chosen processing parameters (grain size, firing temperature, and final sandblasting step) were based on previous investigations by members of our group, demonstrating strong adhesive strength (≈ 70 MPa) and reproducible roughness of glass‑solder coatings on zirconia ceramics [34].

### Sample sterilization

Eleven samples were prepared and handled according to the German version of the EN ISO 10993 standard for biological evaluation of medical devices. Before cell culture experiments, samples were disinfected under aseptic conditions by immersing them in 100% isopropanol in sterile Petri dishes. After a 5-min incubation in isopropanol, the samples were placed on a sterile cloth and air-dried for 10 min.

## Reference materials

Toxic control: A standardized cytotoxic reference material, RM-A (polyurethane film containing 0.1% zinc diethyldithiocarbamate; Hatano Research Institute, Japan), was used as a positive toxic control (per EN ISO 10993).

Non-toxic control: Tissue-culture grade glass coverslips (SARSTEDT, Nümbrecht, Germany; cat. no. 83.1840.002) were used as negative (non-toxic) controls for cell culture assays.

### Cell culture

L929 mouse fibroblasts (LGC Standards, Wesel, Germany) were cultured in Minimum Essential Medium (MEM) supplemented with 10% fetal bovine serum (FBS), 100 U/mL penicillin, 100 μg/mL streptomycin, and 4 mM L-glutamine (Life Technologies, Carlsbad, CA, USA/Sigma–Aldrich, St. Louis, MO, USA). Cells were maintained at 37 °C in a humidified atmosphere of 5% CO₂ (standard cell culture conditions). This complete medium is referred to hereafter as “cell culture medium”. Cells were passaged at ~ 80% confluence for expansion.

HDPCs were obtained from the Department of Oral and Maxillofacial Surgery, University Medical Center Hamburg-Eppendorf as previously described [35]. The cells were initially plated in a small volume (~ 100 µL) of Dulbecco’s Modified Eagle Medium (DMEM) and incubated under standard conditions for two days to facilitate attachment and outgrowth. Subsequently, cells were harvested using 0.05% trypsin–EDTA (Thermo Fisher Scientific, Waltham, MA, USA) and reseeded at a density of 5 × 10^3^ cells/cm^2^ for expansion.

Cytocompatibility was assessed via indirect and direct assays as described below. Indirect extract assays used L929 cells, while direct contact assays (live/dead staining) were performed with both L929 cells and HDPCs (see Sects. "Indirect assays"–"Direct contact assays").

### Indirect assays

#### Extraction of sample media

To prepare extraction media, a base medium was made by mixing 440 mL MEM, 50 mL FBS (to 10% final concentration), 5 mL Pen/Strep (100 U/mL penicillin + 100 μg/mL streptomycin final), and 5 mL L-glutamine stock (4 mM final, noting MEM already contains 2 mM). This complete medium was stored at 4 °C for up to one month and used as needed for extractions. The extraction conditions were chosen to approximate the in vivo physiological environment for implanted biomaterials.

ATZ samples (coated and uncoated) and the toxic control material (RM-A) were submerged in the above cell culture medium at a ratio of 3 cm^2^ sample surface area per 1 mL medium, following EN ISO 10993–5 guidelines. Extractions were carried out for 72 ± 2 h under standard cell culture conditions (37 °C, 5% CO₂). In parallel, fresh cell culture medium (with no sample) was incubated under the same conditions to serve as a negative control extract. After 72 h, the samples were removed and the remaining extraction medium was centrifuged at 14,000 rpm for 10 min to remove any particulates. The supernatants (extracts) were collected and used for cytotoxicity assays.

#### LDH assay

Lactate dehydrogenase (LDH) release was measured to evaluate cytotoxic effects (cell membrane damage) in the extracts. The *LDH-Cytotoxicity Assay Kit II* (BioVision Inc., Milpitas, CA, USA) was used according to the manufacturer’s instructions. In brief, 10 µL of each sample extract (supernatant from Sect. "Extraction of sample media") was incubated with 100 µL of the kit’s reaction mixture for 30 min at room temperature in the dark. After adding the stop solution, absorbance was measured using a multi-well spectrophotometer (ELISA reader) at 450 nm (with a 650 nm reference wavelength). Higher absorbance correlates with greater LDH release, indicating higher cytotoxicity.

#### XTT assay

Cell metabolic activity (as a surrogate for viability and proliferation) was assessed using the *Cell Proliferation Kit II (XTT)* (Roche Diagnostics, Mannheim, Germany). The assay was performed per the manufacturer’s protocol. Briefly, L929 cells were plated in 96-well plates and cultured in the various extracts for 24 h. Next, the XTT labeling reagent was mixed with the electron-coupling reagent (at a 1:50 ratio), and 50 µL of this mixture was added to each well. After 4 h of incubation at 37 °C, 100 µL of the resulting formazan solution from each well was transferred to a new plate, and absorbance was measured at 450 nm (650 nm reference) with a multi-well spectrophotometer. The absorbance values (corrected for background) reflect the number of metabolically active cells.

### Direct contact assays

#### Live/dead fluorescence staining

To directly evaluate cell viability on the materials, L929 cells were seeded directly onto the test samples (coated and uncoated ATZ) in 12-well plates (surface area to volume ratio of 5.65 cm^2^/mL, corresponding to one disk per well with ~ 3 mL of medium). After incubation for 24 ± 2 h under standard conditions, a live/dead staining was performed. Propidium iodide (PI, 50 µg/mL stock in PBS; 60 µL per mL of medium) and fluorescein diacetate (FDA, working solution 20 µg/mL in PBS from a 5 mg/mL acetone stock; 500 µL per mL) were added to each well. After 3 min of incubation at room temperature, the samples were gently rinsed with PBS. The cells on the disks were immediately examined with a fluorescence microscope (Nikon ECLIPSE Ti–S/L100, Nikon GmbH, Düsseldorf, Germany) equipped with filters to simultaneously visualize green (viable cells with FDA) and red (nuclei of dead cells with PI) fluorescence.

#### Osteogenic differentiation assay (HDPCs)

Osteogenic differentiation of human dental pulp cells on the samples was assessed using an OsteoImage™ mineralization assay. HDPCs were seeded onto coated and uncoated ATZ samples (placed in 12-well plates) at an initial density of 2 × 10^4^ cells/mL (1 mL per well). Cells were cultured until they reached ~ 60–70% confluence, then the medium was changed to osteogenic differentiation medium (cell culture medium supplemented with 0.1 µM dexamethasone, 50 µM L-ascorbic acid-2-phosphate, and 10 mM β-glycerophosphate). The cells were maintained in this differentiation medium for 14 days, with media changes twice per week. After two weeks, the cells were stained using the OsteoImage™ Mineralization Assay Kit (Lonza, Walkersville, PA, USA) according to the manufacturer’s instructions to detect calcium deposits (mineralized matrix). Parallel samples were subjected to live/dead staining as described in Sect. "Live/dead fluorescence staining" to ensure cell viability during differentiation.

### Hemocompatibility testing

The blood compatibility of the coated and uncoated ATZ samples was evaluated according to DIN EN ISO 10993–4 (tests for interactions with blood). All steps from blood collection to analysis were completed within two hours to preserve physiological conditions. Whole blood from one healthy human volunteer was drawn into EDTA tubes (for complete blood count analyses) and sodium citrate tubes (for coagulation and hemolysis tests). Two ATZ samples (either both coated or both uncoated) were placed into a sterile 40 cm long polyvinyl chloride (PVC) tube (internal dimensions 3/8″ × 3/32″) coated on the interior with “Ph.i.s.i.o.” (phosphorylcholine-based inert surface; Cormed Medizintechnik GmbH, Rüthen, Germany) to prevent nonspecific plasma protein adsorption and platelet adhesion. Then, 4 mL of freshly collected blood was added to the tube (yielding a surface-to-volume ratio of ~ 3 cm^2^/mL, matching the implant surface area exposure in vivo). The flexible tube was bent into a circular loop, and both ends were sealed with heat-shrink tubing. Tubes containing blood without any sample served as negative controls (baseline blood activity), while tubes containing 0.4 mL blood + 3.6 mL sterile distilled water (to induce osmotic hemolysis) served as positive controls for hemolysis. All tubes were gently rotated in a 37 °C water bath for 60 min (to simulate blood flow and ensure uniform contact). After incubation, blood from each tube was collected for analysis of hematological parameters. *Per* local regulations, no formal ethical approval was required for collecting anonymized blood samples from a volunteer donor for this in vitro compatibility test. All blood analyses were performed using standard clinical laboratory equipment at the Institute of Clinical Chemistry, Transfusion and Laboratory Medicine (BG University Hospital Bergmannsheil, Bochum, Germany).

### Statistical analysis

For XTT- and LDH-assays, data are presented as mean ± standard deviation from two independent experiments with four technical replicates. Statistical comparisons were performed using one‑way ANOVA followed by Tukey’s post‑hoc test. For hemocompatibility tests, results are reported as mean ± standard deviation from two independent experiences and are normalized to the values for uncoated ATZ (which were set as 1.0 or 100% for relative comparisons). Statistical analysis was performed using an unpaired two-tailed Student’s *t*-test for comparisons of two groups and analysis of variance (ANOVA) for comparisons of more than two groups (after confirming normal distribution). *p* < 0.05 was considered statistically significant. Significance levels are indicated as: *p* < 0.05 (*), p < 0.01 (**), p < 0.001 (***), *p* < 0.0001 (****).

## Results

**Fig. 1:**
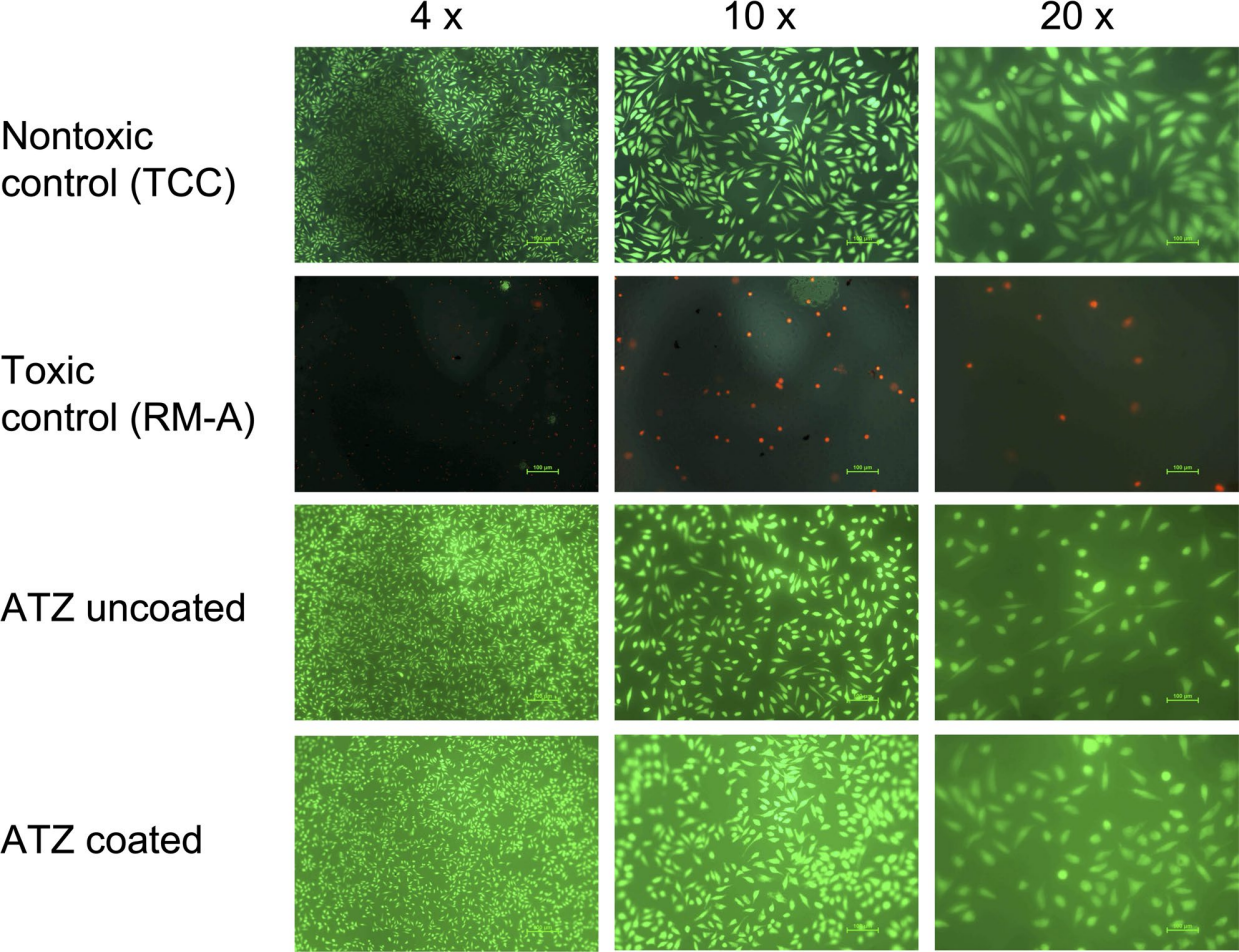
Live/dead staining of L929 mouse fibroblasts. Viable cells, cultured directly on the tested materials, exhibit green fluorescence after staining with fluorescein diacetate. Nuclei of non-viable cells show red fluorescence after staining with propidium iodide. Representative images from two independent experiments are shown at 4×, 10×, and 20× magnification; scale bars represent 100 µm

For direct cytocompatibility and cytotoxicity assessment, L929 fibroblasts were cultured directly on the coated and uncoated ATZ samples and evaluated by live/dead staining. Fluorescence microscopy showed abundant viable cells with healthy spindle-shaped morphology characteristic of fibroblasts on both coated and uncoated surfaces. No dead cells were observed on the surface of either sample type, indicating good cytocompatibility (Fig. 1). The positive and negative control materials produced the expected outcomes, confirming that the assay was working correctly (i.e., the toxic control caused cell death, and the inert control supported cell survival).

**Fig. 2:**
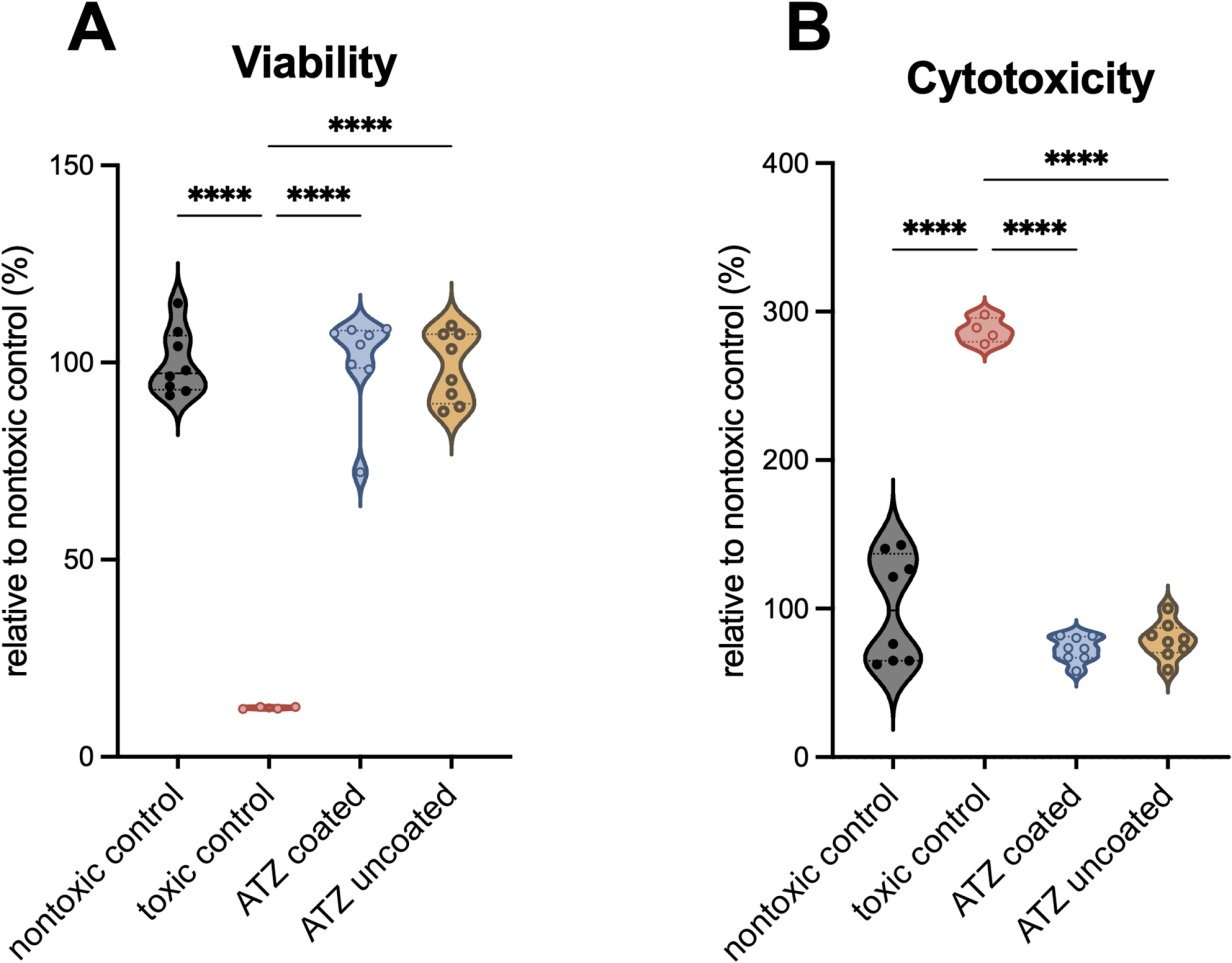
Indirect extraction analysis using L929 mouse fibroblasts. Test samples and the toxic control were incubated in cell culture medium for 72 h. The resulting extracts were then applied to L929 cells and incubated for an additional 24 h. Cell viability was assessed using the XTT assay (**A**), and cytotoxicity was evaluated using the LDH assay (**B**). Level of significance: *p < 0.05, **p < 0.01, ***p < 0.001, ****p < 0.0001

In the indirect extraction assays with L929 fibroblasts (XTT and LDH tests on sample extracts), both the glass‑solder‑coated and uncoated ATZ samples showed excellent cytocompatibility. There were no statistically significant differences between coated and uncoated samples or the negative control extract. Cell viability in all groups exceeded 70% of the negative control, meeting the ISO 10993‑5 criteria for non‑cytotoxicity (Fig. 2). LDH release remained low and comparable across sample types, indicating minimal membrane damage. Although mean values suggested slightly higher metabolic activity and marginally lower LDH levels for the coated samples, these differences did not reach statistical significance and should therefore be interpreted as natural variation within the dataset rather than evidence of an enhanced biological effect.

**Fig. 3:**
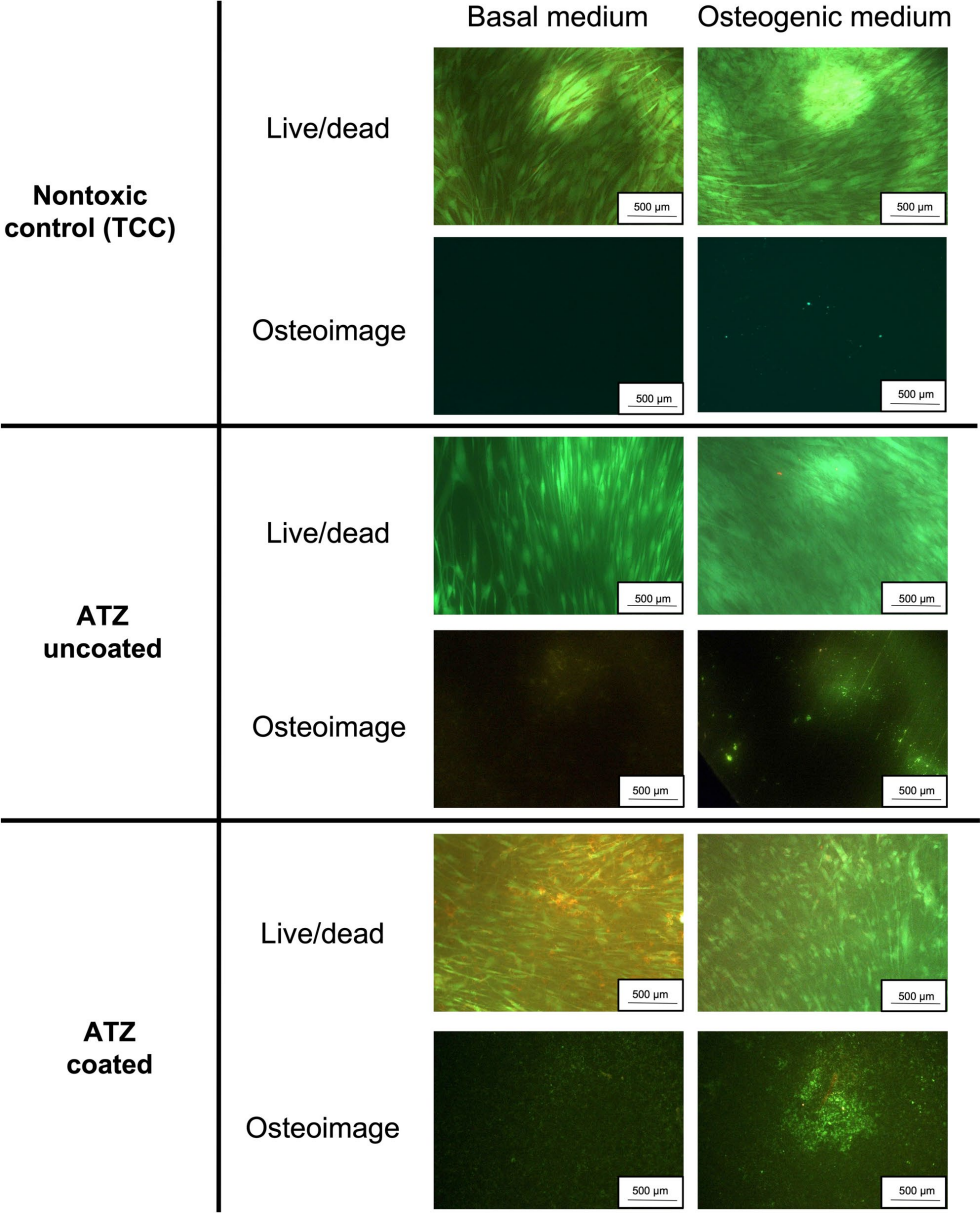
Osteogenic differentiation of human dental pulp cells. Upper panels show live/dead staining: viable cells display green fluorescence after fluorescein diacetate staining, while nuclei of dead cells appear red after propidium iodide staining, indicating no detectable toxic effects. Lower panels show green fluorescence of mineralized matrix structures stained with the OsteoImage™ reagent. Representative images shown from two independent experiments were acquired at 10 × objective magnification

Subsequently, differentiation assays and OsteoImage staining with primary HDPCs showed a good cell response on both the coated and the uncoated ATZ samples. No toxic effects were found in the live/dead staining, neither in the basal medium nor in the osteogenic differentiation medium indicating good properties for the differentiation of osteoblasts (see Fig. 3). No differences in staining intensity or distribution were evident between coated and uncoated surfaces. These findings indicate that the glass‑solder coating did not impair the osteogenic differentiation capacity of HDPCs under the experimental conditions. Because only qualitative methods were applied in this study, no quantitative conclusions can be drawn regarding potential differences in osteogenic activity between the groups.

**Fig. 4:**
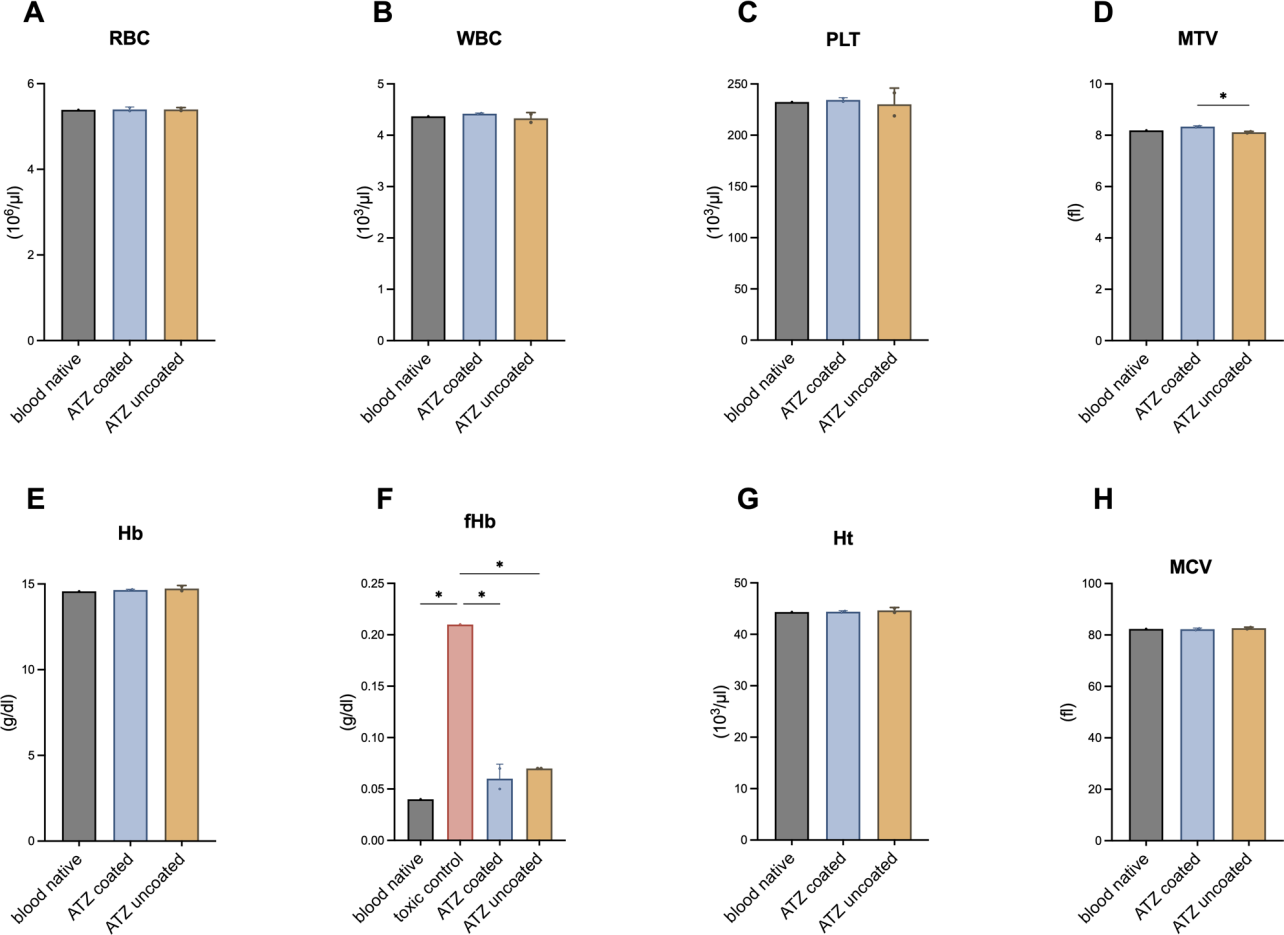
Hemocompatibility testing. Native human blood, uncoated ATZ, and glass-solder–coated ATZ samples were used. For evaluation of free hemoglobin, distilled water was employed to induce cell lysis. The following hematological parameters are shown: **A** red blood cell count, **B** white blood cell count, **C** platelet count, **D** mean platelet volume, **E** hemoglobin concentration, **F** free hemoglobin, **G** hematocrit, and **H** mean corpuscular volume. Level of significance: *p < 0.05, **p < 0.01, ***p < 0.001, ****p < 0.0001

The blood compatibility tests (per DIN EN ISO 10993-4) demonstrated that ATZ specimens coated with the glass solder have no adverse effect on human whole blood. In hemolysis assays, neither the coated nor uncoated samples caused hemolysis: free hemoglobin levels in plasma were negligible and comparable to the negative control (native blood with no sample), whereas the positive control (RM-A) caused a pronounced hemolysis response as expected. Likewise, the coated and uncoated ATZ samples had no significant influence on hematological parameters. Red and white blood cell counts, platelet counts, hemoglobin, hematocrit, mean corpuscular volume (MCV), and mean platelet volume (MPV) remained essentially unchanged from baseline (native blood) when blood was incubated with either type of sample (Fig. 4). In summary, the results for coated samples resembled those for uncoated ceramic and the negative control, across all blood compatibility measures. These data indicate that adding the glass solder coating does not compromise hemocompatibility or induce any detectable blood cell damage or aggregation.

## Discussion

The glass solder coating investigated in this study was developed to improve the bone-to-implant contact (BIC) and osseointegration of ceramic implants in the future. Surface material and topography are known to strongly influence cellular responses, and enhancing these could endorse ceramic implants as veritable alternative to titanium implants [30]. The primary aim of this study was to demonstrate the biocompatibility of the glass solder coating and to characterize the cellular response it elicits. To that end, ATZ ceramic samples were coated with the glass solder and tested according to ISO 10993-5/-12 guidelines using L929 fibroblasts and HDPCs and additionally evaluated for osteogenic potential and blood compatibility (ISO 10993-4 criteria). It was chosen not to include acid-etched ceramic samples as a comparison, because previous studies have reported that acid etching does not significantly alter the mechanical properties of glass-coated ceramics or the cellular responses [36, 37]. The approach of this study, of course, cannot fully replicate the complexity of the physiological environment. Despite this limitation, demonstrating robust cell compatibility and lack of toxicity in vitro is an essential first step for any novel biomaterial before progressing to animal or clinical studies. The selection of appropriate cell models is critical when interpreting in vitro findings related to implant success. In the present study, L929 mouse fibroblasts were used in accordance with ISO 10993 requirements to ensure standardized cytotoxicity assessment, while human dental pulp cells (HDPCs) were employed as a complementary, osteogenically competent human model. HDPCs possess mesenchymal stem‑cell‑like characteristics and a well‑documented capacity to differentiate into osteoblast‑like cells, making them suitable for evaluating early osteogenic responses on implant materials. Their neural‑crest origin also reflects the developmental lineage of craniofacial bone and dentoalveolar tissues, providing biological relevance to dental implant applications. Although primary human osteoblasts, mesenchymal stem cells, or macrophages could provide additional perspectives on bone remodeling and immune interactions, the use of HDPCs offered a reproducible and ethically accessible model for this initial in vitro screening. Therefore, the coating can presently be described as osteogenically compatible rather than osteogenically stimulatory. Future studies will include quantitative readouts to clarify whether the glass‑solder surface may exert any modulatory effects on osteogenic pathways. Overall, the cellular response to the glass solder coating was very favorable. Both the direct contact and extract assays showed good cell viability and no cytotoxicity associated with the coated samples. A non-significant trend toward increased metabolic activity (XTT assay) and a small decrease in LDH release was observed. However, these differences did not reach statistical significance and therefore do not indicate an enhanced effect. More importantly, the coated surfaces did not negatively affect osteogenic differentiation of HDPCs: cells on the glass-coated ceramic were able to deposit mineralized matrix similarly to those on uncoated ceramic. This indicates that the coating does not impair osteogenic differentiation and is compatible with osteogenic activity. Markhoff et al*.* reported that roughened ceramic coated with a similar glass solder supported increased metabolic activity of human osteoblasts [38]. Our findings are consistent with previous reports indicating that glass solder coatings are biocompatible and do not adversely affect bone-related cell functions. In addition, previous works have shown that ceramic implant surfaces coated with glass-based materials maintain adequate cytocompatibility with both osteoblasts and epithelial cells, while also possessing sufficient mechanical stability [36, 38]. These concurrent improvements in mechanical and biological properties are encouraging for the future use of such coatings.

Another key requirement for any implant material is blood compatibility, as the implant will contact blood during surgery and healing. The process of blood coagulation can be initiated by a sequence of proteolytic reactions, resulting in the formation of fibrin clots. This process involves both intrinsic and extrinsic coagulation pathways [39]. In our hemocompatibility assessments, the glass solder-coated samples performed equivalently to uncoated zirconia, with no hemolysis and no adverse changes in blood cell counts or indices. This is a pivotal finding because it indicates that the new coating would not be expected to cause thrombogenic or hemolytic complications in a future clinical setting. The lack of difference between coated and uncoated samples also implies that the glass solder itself does not leach any components that trigger blood cell activation or destruction. A limitation of the present hemocompatibility analysis is that blood was obtained from a single healthy donor per test, in line with ISO 10993‑4 recommendations for preliminary in vitro screening. While this approach is appropriate to identify major hemolytic or cytotoxic effects, it does not allow for robust statistical inference across independent biological samples. Accordingly, These analyses should be regarded as descriptive and explorative. Future work will include blood from multiple donors to confirm the reproducibility of the current findings and to allow statistically valid inter‑individual comparisons.

It should be noted that any surface modification can potentially lead to the release of particulate debris at the time of implant placement. Senna et al. showed that altering the implant surface (chemically or topographically) can generate particles of various sizes during insertion into bone [40]. Therefore, a prudent next step would be to assess whether the glass solder coating sheds any particles upon implant insertion and, if so, whether those particles elicit an inflammatory response. In the context of inflammatory mediators, studies comparing titanium and zirconia have found that titanium wear particles provoke significantly higher TNF-α release from macrophages, whereas zirconia particles have minimal effect on TNF-α levels [24]. If the glass solder coating adheres well and minimizes particulate release, it could help ceramic implants remain immunologically favorable. Ensuring strong adhesion of the coating (to avoid delamination or chipping) will be as important as the in vitro biocompatibility which was demonstrated in this study. A further translational aspect concerns the potential release of particulate debris from the coated surface during implant insertion. This issue is especially relevant for coatings produced by multi‑step spray‑coating and sintering procedures, where local variations in layer thickness or adhesion strength might occur. In the present study, no experimental analysis of coating integrity or particulate release was performed, representing an important limitation. Future investigations will therefore include quantitative surface characterization using scanning electron microscopy, profilometry, and adhesion testing, as already established for comparable glass‑solder coatings on zirconia substrates [34].

Recent advances in laser‑based structuring of glass‑solder‑coated zirconia surfaces have further highlighted that processing parameters—such as laser fluence and scanning speed—can substantially influence coating morphology, roughness, and wettability [41]. These complementary findings underline the importance of correlating structural properties (Ra/Sa values, coating thickness, and uniformity) with biological outcomes in future work.

Furthermore, quantitative osteogenic endpoints (alkaline phosphatase activity, Alizarin Red stainging, gene expression analyses) will be investigated to confirm osteogenic differentiation. In addition, simulated insertion experiments under standardized torque conditions will be planned to assess whether the glass solder layer remains intact or generates debris. These data will be essential to validate the translational safety of the coating prior to in vivo application.

Our findings highlight another consideration in implant surface engineering: current methods (grit-blasting, etching, plasma-spraying, dip-coating, etc.) produce surfaces that, while roughened, are essentially stochastic in their architecture. Cells may prefer more ordered micro-scale patterns [31]. Thus, future research should aim to develop surface modification techniques that yield controlled, reproducible micro topographies with uniform layer thickness. One promising approach is computer-aided inkjet printing using a glass solder suspension as an ink to create precise 3D microstructures on the implant surface [42].

Finally, beyond ceramic implants, it is worth considering hybrid implant designs. For example, Ti implants could be coated with a biocompatible glass solder layer. Such a hybrid implant might combine the structural resilience of Ti with the excellent biocompatibility of the ceramic-like glass surface. This concept remains explorative at this stage; further investigations would be needed to evaluate the mechanical behavior of a glass-coated Ti implant and its biological performance in vivo. Nonetheless, it represents an exciting avenue for future innovation in implantology.

## Conclusions

Innovative biomaterials for implants must meet key requirements related to their potential clinical application, including biocompatibility and the ability to support tissue integration. Surface modification by the glass solder tested demonstrated favorable cytocompatibility and hemocompatibility, and represents a biocompatible surface modification approach for further investigation. No evidence of toxicity was observed in any of our assays. These results suggest that the glass solder-coated ceramic is a promising candidate for improving dental implant surface and biological response. In vivo studies and long-term investigations will be the next steps to confirm its safety, efficacy and function in a clinical context.

## Data Availability

The datasets used and/or analyzed during the current study are available from the corresponding author on reasonable request.

## Abbreviations

ALP: Alkaline Phosphatase
ATZ: Aluminum Toughened Zirconia
BIC: Bone-to-Implant Contact
DMEM: Dulbecco’s Modified Eagle Medium
ELISA: Enzyme-Linked Immunosorbent Assay
FBS: Fetal Bovine Serum
FDA: Fluorescein Diacetate
HDPC: Human Dental Pulp Cells
IL-1: Interleukin-1
LDH: Lactate Dehydrogenase
PI: Propidium Iodide
PVC: Polyvinyl Chloride
RANKL: Receptor Activator of NF-κB Ligand
RM-A: Reference material-A (toxic control)
TCC: Tissue culture coverslips (non-toxic control)
Ti: Titanium
TNF-α: Tumor Necrosis Factor alpha
